# Dataset on the durability behavior of palm oil fuel ash self compacting concrete

**DOI:** 10.1016/j.dib.2018.05.121

**Published:** 2018-05-24

**Authors:** O.M. Ofuyatan, S.O. Edeki

**Affiliations:** a Department of Civil Engineering, Covenant University, Ota, Nigeria; b Department of Mathematics, Covenant University, Ota, Nigeria

**Keywords:** Permeability, Durability, Self compacting concrete, Sulphate resistance, Palm ash, Acid attack

## Abstract

Datasets contained in this article are outcomes of the durability properties carried out on Palm Oil Fuel Ash (POFA) concrete specimens to determine the effect of acid attack, Sulphate resistance, water absorption and rapid chloride permeability. Specimens were immersed in 1% hydrochloric acid (H_2_SO_4_) solution, 5% sodium Sulphate for 28, 56 and 90 days and different exposure conditions. The dataset helps the readers to understand and evaluate the potential of POFA though a waste material can be used as a replacement of Portland cement in having durability properties at an acceptable range.

**Specifications Table**

**Table:** 

Subject area	*Civil Engineering*
More specific subject area	*Concrete production and durability*
Type of data	*Table, figures.*
How data was acquired	*Laboratory experiment (control)*
Data format	*Raw and Analysed*
Experimental factors	*Concrete durability and permeability properties*
Experimental features	*Exposure of coefficient of water permeability for specimens*
Data source location	*Experimental and laboratory*, Nigeria
Data accessibility	*Data are within this article.*

**Value of the data**

• The data contained herein can be used to solve some of the problems of durability of concrete in the cement industries.
• The data can be used to determine durability behaviour of self compacting concrete (SCC) [1].
• The employed approach can serve as benchmark to other methods in testing properties of SCC.
• The dataset can be used to determine performance required for concrete structure.
• The dataset can serve as an experimental framework for the analysis of other basic properties of concrete.

## 1 Data

**Fig. 1:**
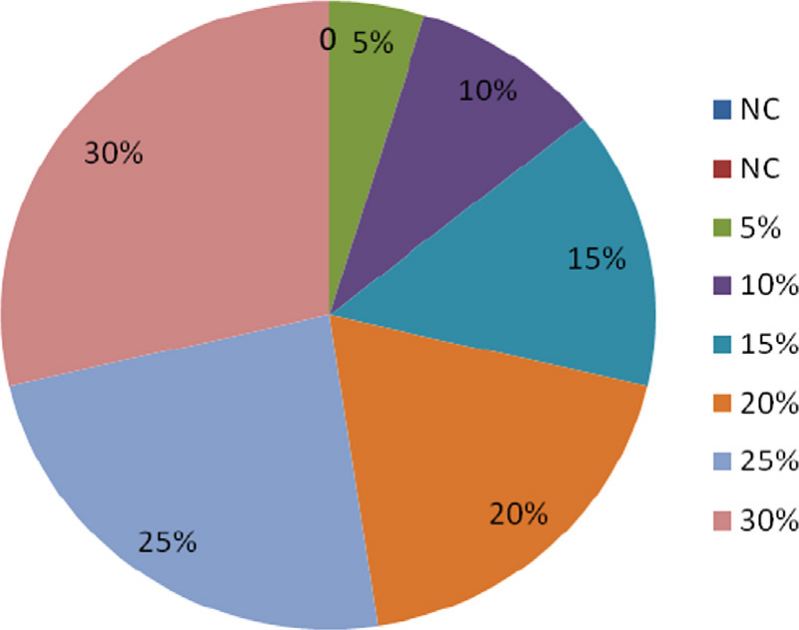
Weight loss vs varying percentages of POFA.

**Fig. 2:**
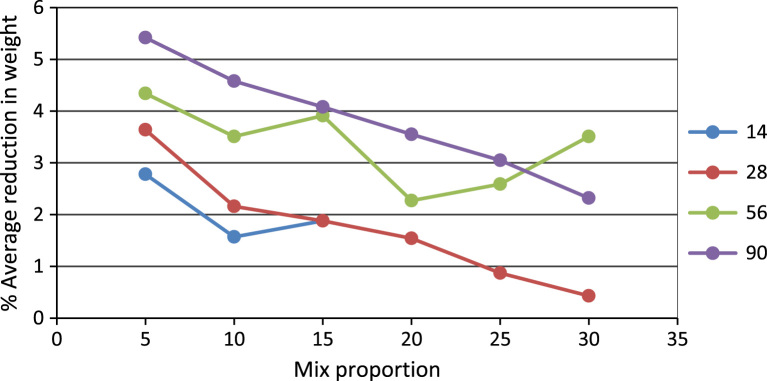
Average reduction in weight.

**Fig. 3:**
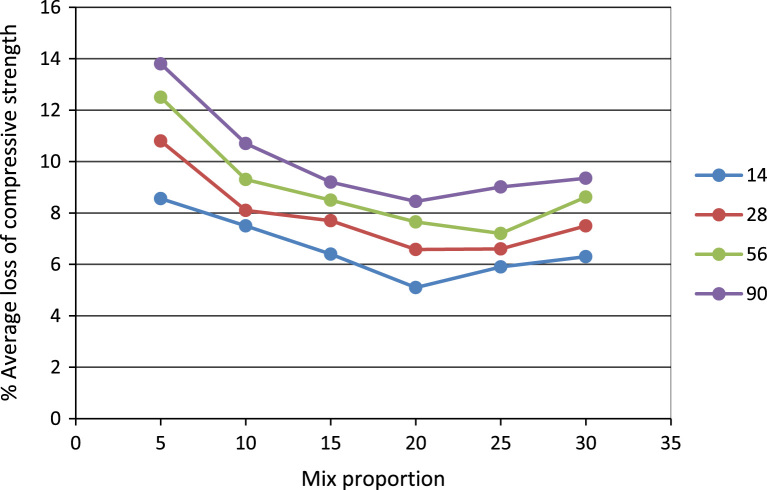
Average loss of compressive strength.

**Fig. 4:**
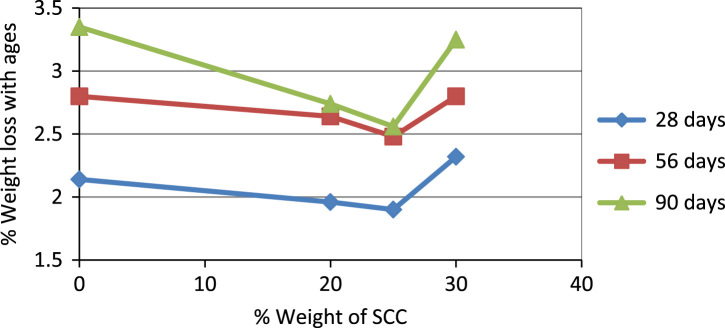
Sulphate attack test results.

**Fig. 5:**
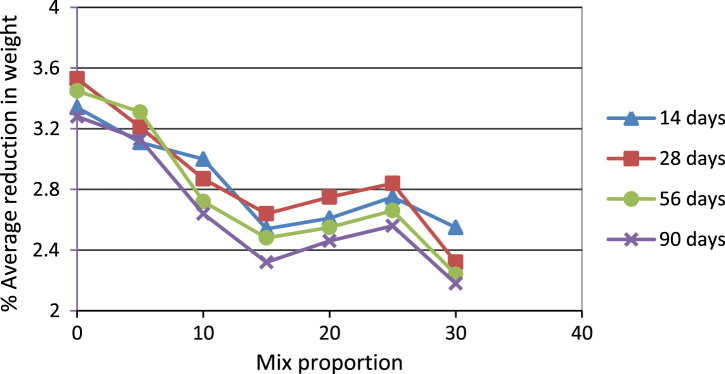
Water absorption rate test results.

**Fig. 6:**
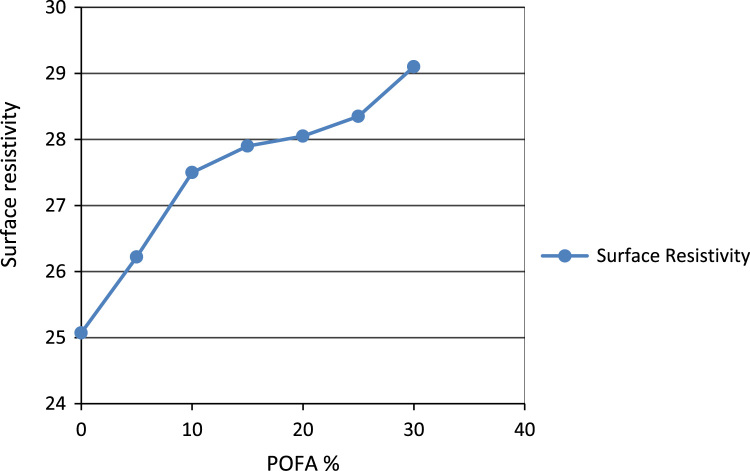
Concrete surface resistivity test results.

In this dataset article, durability test, acid attack, sulphate, water absorption test, rapid chloride permeability test on POFA self-compacting concrete test were carried out while the test results are as contained in Table 1, Table 2, Table 3, Table 4, Table 5, and Fig. 1, Fig. 2, Fig. 3, Fig. 4, Fig. 5, Fig. 6 are as follows. For more clarification and enhancement of the paper quality, better representations of the information contained in Table 1 are presented in Figs. 2 and 3. These contain the plots of the mix proportion vs the % average reduction in weight (Fig. 2), and mix proportion vs % average loss of compressive strength (Fig. 3). In addition, Fig. 4, Fig. 5, Fig. 6 are presented to buttress the points contained in Table 3, Table 4, Table 5. Readers are referred to [2], [3], [4], [5], [6], [7], [8], [9], [10], [11] for related references.

**Table 1:** Results of the weight loss in acid attack.

No	Concrete type	Weight loss in acid attack (90days)
1	NC not vibrated	−15.17
2	NC vibrated	−4.30
3	SCC 5%	−20.73
4	SCC 10%	−11.09
5	SCC 15%	−23.12
6	SCC 20%	−28.16
7	SCC 25%	−37.86
8	SCC 30%	−57.29

**Table 2:** Acid resistance test results.

Mix Proportion	Average reduction in weight %				Average loss of compressive strength %			
**–**	14	28	56	90	14	28	56	90
5	2.78	3.64	4.34	5.42	8.56	10.8	12.5	13.8
10	1.57	2.16	3.51	4.58	7.5	8.1	9.3	10.7
15	1.88	1.88	3.91	4.08	6.4	7.7	8.5	9.2
20	1.54	1.54	2.27	3.55	5.1	6.58	7.65	8.45
25	0.87	0.87	2.59	3.05	5.9	6.6	7.20	9.01
30	0.43	0.43	3.51	2.32	6.3	7.5	8.62	9.35

**Table 3:** Sulphate attack test results.

	% weight loss with ages		
%SCC	28 days	56 days	90 days
0	2.14	2.8	3.35
20	1.96	2.64	2.74
25	1.9	2.48	2.56
30	2.32	2.8	3.25

**Table 4:** Water absorption rate test results.

Mix proportion	Average reduction in weight %			
	14	28	56	90
0	3.34	3.53	3.45	3.28
5	3.11	3.21	3.31	3.13
10	3.00	2.87	2.72	2.64
15	2.54	2.64	2.48	2.32
20	2.61	2.75	2.55	2.46
25	2.75	2.84	2.66	2.56
30	2.55	2.32	2.24	2.18

**Table 5:** Concrete surface resistivity test results.

POFA %	Surface Resistivity	Chloride ion Penetrability
0	25.07	Low
5	26.22	Low
10	27.50	Low
15	27.90	Low
20	28.05	Low
25	28.35	Low
30	29.1	Low

## 2 Experimental design, materials and methods

### 2.1 Data analysis with respect to acid resistance

The results of acid attack in terms of weight loss after 90 days for all the concrete are shown in Table 1. It can be seen that the concrete of lower strength, the weight loss decreased with increasing percentage of palm ash replacement. But concretes of higher strengths showed a marginal higher weight loss particularly at higher exposure times. This was probably due to the fact that the acid attack as is known is primarily related to the actual cement contents in these concrete. The weight loss was studied in terms of cement contents replacement. The relationship between weight loss at 90 days is shown in Fig. 1 and cement content in Table 2.

### 2.2 Data analysis with respect to Sulphate Attack results

External sulphate attack to cementious matrix is a complex process that involves the movement of sulphate ions through the pores by means of different mechanisms of transportation and the interaction of aggressive solution with some compounds of cements paste to form expansive compounds (gypsum) that produce cracking, strength loss and softening. After 28 days of curing the test was conducted by measuring the weight losses of the specimen at 28, 56 and 90 days. Table 3 shows the percentage loss in weight of concrete specimens made by using various amount of replacement in cement quantity by palm ash with respect to age.

### 2.3 Data analysis with respect to Water absorption test results

The result of Saturated Water Absorption (SWA) tests of various self-compacting concrete mixes at the ages of 28, 56 and 90 days are given in Table 4. It was noted that with the increase in Palm ash content the saturated water absorption gets decreased when compared with the control mix. Palm ash acts as a filter material which fills the pores and thereby reduces water absorption.

### 2.4 Data analysis with respect to rapid chloride permeability test

Table 5 shows the results of the chloride penetration. The resistance to such diffusion can be increased by refining the pore-structure of the concrete. The implications of such substantial decreases in chloride ion penetrability can be considered in the design of offshore structures, bridge decks, parking garages and other structures that are vulnerable to corrosion of reinforcing steel under chloride ion attack.

In the text, SCC and NC denote self compacting concrete and normal concrete (without POFA) respectively.

It was observed that the incorporation of palm oil fuel ash in self-compacting concrete enhanced the Sulphate and acid attack. The rapid chloride and water absorption showed a reduction in weight. It can also be seen that both normal concrete as well as SCC palm ash have all been following the same trend. In Table 3, it is observed that with the increase in palm ash content, the weight reduction gets decreased when compared with the control mix. It is clear that palm ash added as partial replacement in concrete enhances the sulphate resistance in concrete.
